# Broadening global health knowledge: epistemic pluralism through Asian philosophical traditions

**DOI:** 10.7189/jogh.16.03038

**Published:** 2026-11-13

**Authors:** Bhadrajee Hewage, Bishal Joshi, Yogesh Poudyal, Shuma Banik, Borimas Hanbookunuparkarn, Haruhiko Ishioka, Junjie Ding, Sanjaya Acharya, Shiva Raj Mishra, Bipin Adhikari

**Affiliations:** 1 Department of History, University of Oxford, Oxford, UK; 2 Department of Physiology, Universal College of Medical Sciences, Bhairahawa, Nepal; 3 Sarnath Skin Centre, Bhairahawa, Nepal; 4 Centre for Tropical Medicine and Global Health, Nuffield Department of Medicine, University of Oxford, Oxford, UK; 5 Mahidol-Oxford Tropical Medicine Research Unit, Bangkok, Thailand; 6 Division of Infectious Diseases, Jichi Medical University Hospital, Tochigi, Japan; 7 Academy for Data Science and Global Health, Kathmandu, Nepal

**Keywords:** global health, orientalism, empathy, knowledge pluralism, Asian philosophy

## Abstract

Global health continues to operate largely within a biomedical epistemological framework that, while effective in addressing acute disease and advancing clinical care, remains limited in responding to complex challenges such as mental health, chronic illness, and persistent inequities. These limitations are not merely technical but reflect deeper epistemic constraints in how health and care are conceptualised. This article argues that selected Asian philosophical traditions, often interpreted through the lens of orientalism, offer an under-recognised resource for rethinking global health. Drawing selectively on Taoist, Confucian, and Buddhist traditions, we highlight how principles of relationality, adaptability, and compassion provide alternative foundations for understanding health beyond the absence of disease. These traditions offer distinctive conceptualisations of relationality, compassion and well-being that can be placed in dialogue with contemporary biomedical and public-health approaches. While contemporary global health systems have increasingly incorporated practices such as mindfulness-based interventions, this integration has often occurred without engagement with their broader philosophical and epistemological implications. Recognising these perspectives and their potential for integration has important implications for global health practice, including reorienting medical education toward reflective and relational competencies, strengthening person-centred and community-engaged care models, and addressing knowledge hierarchies that marginalise ancient philosophical traditions. Reimagining global health therefore requires moving beyond unidirectional knowledge transfer toward a more pluralistic and dialogic framework, in which diverse traditions contribute to more holistic, equitable, and contextually responsive health systems.

Orientalism was often the primary lens through which the West looked at Eastern philosophies, principles and knowledge capital to inform and shape existing worldviews [1,2]. In its traditional sense, Orientalism is a Western frame of looking at Eastern knowledge [2,3]. In recent years, a renewed interest in knowledge pluralism has prompted a re-examination of ancient knowledge traditions that once guided early civilisations [4].

Lao Tzu, the founder of Taoism, uses the metaphor of a river, like a property navigating through the harshness of impenetrable rocks and invincible mountains. For humanity, this may translate to redirecting oneself toward what is possible, rather than radically and linearly pushing through obstacles. This is further reflected in the principle of *Wu wei*, in which effective action arises not from forceful resistance, but from aligning oneself with the natural flow of circumstances and responding to them without contrivance [5,6]. Confucianism offers another example of how self-effacement and humility offer one a powerful weapon to achieve one’s objective [7]. Buddha – the light of Asia offered a sense of purpose through introspection, finding peace in self by disengaging from materialism and ego-centrism [8]. Selected philosophical traditions originating in Asia offer concepts that may contribute to contemporary discussions of reflection, compassion, relationality and well-being in global health [9].

Global health has immense potential to embrace Asian philosophies, and ultimately devise collective solutions irrespective of borders (geography, race, creed, and wealth) [10,11]. Global health efforts should thus transcend boundaries and borders to safeguard the vulnerabilities of all populations. Such a level of collective effort is only feasible when the concept of ‘solidarity’ is embraced, which explains why and how we should behave as ‘one’ for all populations [12]. This particular concept emanates from the human neuro-behavioural response, particularly embedded in ‘empathy’ [13].

In the context of global health, advancing the discourse on Asian philosophies also addresses knowledge hierarchy that favours biomedical knowledge over ancient knowledge systems, often treated as complementary rather than foundational [14]. Recognising these knowledge asymmetries is pivotal as global health systems increasingly operate across diverse cultural and philosophical traditions [15,16]. It also offers an opportunity to broaden the epistemological foundations of contemporary global health. This argument is not intended to idealise Asian philosophies, nor to reject biomedical science. Asian philosophies themselves are heterogeneous, historically situated, and sometimes implicated in hierarchy or exclusion. The argument instead is for epistemic plurality and dialogue to foster harmony and respect across knowledge systems rather than their replacement.

Here, we examine how selected concepts from Asian philosophical and medical traditions could contribute to epistemic pluralism in global health, using mindfulness, relationality and compassion as illustrative examples.

## Reflexivity

BH is a scholar of Sri Lankan origin who was raised primarily in Ireland, with a specialisation in the history of Buddhism. BJ is a clinical physiologist who attained all his education in Nepal and is undertaking a PhD on the impact of Vipassana meditation. YP is a clinician who completed all his education in Nepal, with interests and scholarly engagement in Buddhist and Eastern philosophies. SB is an Indian public health researcher based at Oxford, with interests in Eastern philosophy and knowledge pluralism. BH is a Thai physician with expertise in Tropical Medicine and Buddhist literature and practice. HI is a Japanese physician and tropical medicine expert currently practising in Japan. JD is a Chinese pharmacologist who attained all his education in China and was based at the Mahidol-Oxford Tropical Medicine Research Unit with expertise in pharmacological interactions of antimalarial drugs in the human body. SA is a Nepalese physician with interests and scholarly engagement in Eastern philosophical literature. SRM is a Nepalese public health expert who engages with global health literature, particularly emphasising the concept of epistemic indebtedness – a concept that calls for active contributions of knowledge from the Global South. BA grew up and attained his primary education in Nepal, China, Thailand, and the UK, and engages with global health literature, advocating for knowledge pluralism.

## ASIAN PHILOSOPHICAL PERSPECTIVES ON GLOBAL HEALTH

To situate the argument more clearly for the objective, it is useful to contrast the dominant biomedical paradigm in global health with key principles drawn from Asian philosophical traditions. While the former has prioritised disease-centred and at times reductionist approaches, the latter emphasises relationality, adaptability, and compassion as foundational to health and care. These contrasts are not merely philosophical but have practical implications for how global health systems can be conceptualised and reformed (**Table 1**).

**Table 1:** Key contrasts between dominant biomedical paradigms and selected Asian philosophical perspectives in global health

Dimension	Contemporary global health	Illustrative concepts from selected Asian philosophical traditions	Potential areas for dialogue
Concept of health	Health as absence of disease	Health as harmony (mind–body–spirit–environment)	Shift toward holistic and preventive care
Approach to illness	Reductionist, disease-focused	Contextual, relational, systems-oriented	Better management of chronic and syndemic conditions
Role of empathy	Communication skill	Core ethical and structural principle	Strengthens trust, adherence, and care quality
Patient role	Some degree of passivity among patients as care recipients	Emphasis on active role of participant in care and healing	Supports person-centred and community-based care
Knowledge systems	Dominated by Western epistemology	Pluralistic, rooted in diverse traditions	Promotes epistemic equity and inclusivity
Mental health	Symptom management	Integration of awareness, reflection, and balance	Enhances resilience and preventive mental health
Healthcare delivery	Institutional, specialised	Integrative, community-engaged	Bridges clinical care with social and behavioural health
Global health model	Knowledge transfer (North → South)	Epistemic dialogue and knowledge pluralism	More equitable global health governance

### Holistic global health

Several medical and philosophical traditions originating in Asia, including Ayurveda, traditional Chinese medicine, Tibetan medicine and Buddhist traditions, contain accounts of health that extend beyond disease pathology to relationships among bodily, psychological, social, environmental and, in some traditions, spiritual dimensions of well-being [17–19]. Although Asian health philosophies entail a heterogeneous set of medical texts and traditions, such as the Ayurvedic *Charaka Samhita*, traditional Chinese, Greco-Arabic texts within Unani medicine, Tibetan, and Buddhist medical writings, they have remarkable commonalities in their holistic understanding of health (**Box 1**; File S1 in the **Online Supplementary Document**). Ayurveda, for instance, focuses on balancing dietary and lifestyle factors, while Traditional Chinese medicine focuses on balancing energy in the human body by harmonising the energy channels through herbs, acupuncture, acupressure and exercises [20]. Similarly, Unani medicine highlights the balance of humours through diet, regimen, and natural therapies, and Tibetan medicine integrates bodily balance with mental and spiritual harmony through diet, behaviour, and medicinal formulations. At the same time, Buddhist practices of equanimity of mind, ethical living and mindfulness meditation impact physical, mental and emotional well-being.

Box 1 **Glossary of key terms used in this article**

**Term and a brief meaning**
**Orientalism:** a traditional system of thought and representation, developed largely in the West, that constructs ‘the East’ as fundamentally different, inferior, and knowable in ways that justify and sustain cultural, political, and economic dominance.
**Occidentalism:** refers to the set of representations and critiques that construct ‘the West’ as a coherent cultural and political entity, often portraying it as materialistic or hegemonic and often appearing within Western self-critique.
**Taoism:** a Chinese philosophical and religious tradition centred on the Dao (the ‘Way’) as the ineffable, generative principle underlying all reality, teaching that human flourishing arises from aligning oneself with its spontaneous processes.
**Wu wei:** a Taoist principle of non-coercive action in which one acts in complete alignment with the Dao, allowing outcomes to arise spontaneously without force, strain, or contrived intention.
**Confucianism:** a Chinese ethical and philosophical tradition, associated with Confucius, that centres on the cultivation of moral character and the proper ordering of society through relational virtues, ritual practice, and the ideal of humane governance.
**Buddhism:** refers to a religious and philosophical tradition founded by Gautama Buddha that teaches that existence is marked by suffering, caused by craving and ignorance, and that liberation is achieved through insight into reality and disciplined practice along the Noble 8-fold Path.
**Anicca:** the Buddhist doctrine that all conditioned phenomena are inherently transient, arising and passing away moment by moment, and that insight into this impermanence undermines attachment and is essential for liberation.
**Anattā:** the Buddhist doctrine that no permanent, independent self exists; what we take to be a ‘self’ is instead a contingent aggregation of changing physical and mental processes, and insight into this dissolves attachment and supports liberation.
**Sati:** the cultivated capacity for lucid, continuous awareness that ‘remembers’ to attend to present experience clearly and without grasping, forming a core component of meditative practice and the path to insight.
**Vipassanā:** refers to a form of Buddhist meditation that systematically cultivates direct insight into the nature of experience, especially impermanence, suffering, and non-self through sustained, mindful observation of bodily and mental processes, leading toward dispassion and liberation.
**Mettā:** a cultivated attitude of boundless, non-possessive goodwill that actively wishes the welfare and happiness of all beings, developed through meditative practice as a stabilising force that counteracts aversion and supports ethical conduct and liberation.
**Karuṇā:** the cultivated quality of compassionate responsiveness that arises from perceiving the suffering of beings and expresses itself as the sincere intention and readiness to alleviate that suffering, developed through meditative practice and ethical discipline.
**Prakṛti:** refers to a person’s natural, inborn body–mind constitution in Ayurveda
**Dharma:** a foundational concept in Buddhism referring to the truth or law governing reality, the Buddha’s teachings that articulate this truth, and the ethical and meditative path through which it is realised and embodied.
**MBSR:** Mindfulness-Based Stress Reduction, a structured mindfulness programme used in healthcare.
**MBCT:** Mindfulness-Based Cognitive Therapy, a mindfulness-based intervention used especially for depression relapse prevention.
**Syndemic:** two or more interacting health problems that worsen each other in a social and structural context.

Biomedical approaches have generated major advances in diagnosis, treatment, surgery and disease prevention. However, when applied narrowly, biomedical models may privilege measurable pathology and individual-level mechanisms over relational, social, environmental and experiential dimensions of health. These limitations have also been recognised within contemporary medicine itself, including through biopsychosocial, person-centred and social-determinants approaches [21]. While scientific approaches have traditionally emphasised health as the absence of disease, the World Health Organization broadens this view by defining health as a state of complete physical, mental, and social well-being, not merely the absence of disease or infirmity [22]. The biomedical approach is indeed effective in managing acute and emergency conditions, surgical interventions, and disease prevention through vaccines. However, it overlooks the human body’s elemental connection to nature, time and space, and the prominent mental, social and preventive roles in health. For example, the biomedical model generally considers illness only when identifiable pathology is present. Although psychosomatic disorders are now increasingly recognised in Western medicine, Eastern medical traditions have long emphasised that many physical illnesses originate from disharmony between body, mind and spirit [23-26]. There is an oversight in present global health approaches, which ignore sub-clinical nuances of well-being.

### Mindfulness and mental health

Mindfulness has roots in Buddhist philosophy extending back more than 2500 years [27]. Contemplative practices are also found in other Eastern traditions. Hindu traditions, for example, describe meditation in the *Yoga Sutras* and *Advaita Vedanta*, while Zen traditions in China and Japan developed forms of seated meditation centred on attentive observation without attachment or judgement [28].

The incorporation of mindfulness into contemporary healthcare provides a useful example of how an Eastern contemplative practice has been adapted within Western clinical settings. Research has associated mindfulness-based interventions with improvements in stress, anxiety, pain, and other psychological and physical symptoms [29,30]. Two prominent clinical adaptations are Mindfulness-Based Stress Reduction (MBSR) and Mindfulness-Based Cognitive Therapy (MBCT) [31]. Developed in 1979, MBSR adapted Buddhist-derived contemplative practices for use within medicine and psychology and has subsequently been applied across a range of physical and mental health conditions [31–33]. MBCT later combined mindfulness practice with elements of cognitive therapy, particularly for preventing relapse in recurrent depression [34].

The relevance of mindfulness to the present argument therefore extends beyond its clinical effects. Its incorporation into contemporary healthcare illustrates how practices derived from Eastern knowledge traditions can be translated into evidence-based interventions within biomedical systems. At the same time, such translation raises a broader question for global health: whether useful practices should be adopted in isolation, or whether greater attention should also be given to the philosophical and ethical traditions from which they emerged.

### Buddhist psychological concepts and contemporary psychology

Buddhist psychology and contemporary clinical psychology developed in different historical and intellectual settings, but they overlap. Buddhist approaches have traditionally been concerned with understanding and reducing suffering through changes in awareness, conduct, and ways of relating to experience [35,36]. Contemporary psychology addresses suffering through a wide range of approaches, including symptom-focused treatment as well as preventive, behavioural, relational, and well-being-oriented models. Rather than treating these traditions as opposites, several Buddhist concepts can be considered alongside related ideas in contemporary psychology.

### *Anicca* (impermanence) and cognitive flexibility

One such concept is *anicca*, or impermanence: the Buddhist view that physical and mental experiences continually change [37]. Recognising this transience is intended to reduce attachment to experiences as though they were fixed or permanent. A loose parallel can be found in the psychological concept of cognitive flexibility – the capacity to adapt one’s thinking or behaviour when circumstances change [38]. Research on mindfulness has associated meditation practice with measures of attentional control and cognitive flexibility [38,39]. These concepts are not equivalent, but their comparison illustrates how an older Buddhist account of responding to change can enter into dialogue with contemporary psychological approaches to adaptation.

### *Anatta* (non-self) and decentring

A second concept, *anattā* or non-self, questions the idea of the self as a fixed and unchanging entity [40]. Buddhist accounts instead describe experience as arising through changing physical and mental processes [41]. A related, although distinct, concept in contemporary psychology is decentring: the ability to recognise thoughts and feelings as mental events rather than automatically treating them as facts or as defining features of oneself [42]. Decentring has been associated with better psychological outcomes in research on mindfulness-based interventions [43]. *Vipassanā* (insight) meditation similarly encourages observation of sensations, thoughts, and emotions without identifying with them as permanent features of the self [44]. The *Anattalakkhaṇa Sutta* provides an early textual basis for this Buddhist account of non-self, stating:

Form, feeling, perception, volitional formations, and consciousness are not-self. Were they self, they would not lead to affliction, and one could say, ‘Let my body be thus, let my mind be thus.’ But because they are not-self, they lead to affliction, and none can control them. *[**45**]*

### *Sati* (mindfulness) and meta- awareness

*Sati*, commonly translated as mindfulness, refers in Buddhist practice to sustained awareness of present experience [46]. *Vipassanā* develops this capacity by encouraging observation of bodily sensations, thoughts, and emotions as they arise and pass [44]. Contemporary psychology uses the related concept of meta-awareness to describe the capacity to notice one's own thoughts and attentional states [46]. Experimental research suggests that mindfulness training can reduce mind-wandering and improve attentional monitoring [47], while neurocognitive models have proposed mechanisms through which mindfulness may support self-awareness and self-regulation [48]. Although the Buddhist and psychological concepts arise from different traditions and should not be treated as interchangeable, these parallels show how Buddhist ideas have contributed to contemporary discussions of attention and psychological well-being.

### Compassion and the practice of care

The relevance of these traditions to healthcare extends beyond mindfulness. Some Ayurvedic and Buddhist traditions place particular emphasis on the moral qualities of the practitioner and on compassionate relationships with those who are suffering. In Ayurvedic literature, *karuṇā* (compassion) and *sattva* (clarity of mind) are associated with the conduct of the healer [49]. The concept of *prakṛti*, referring to an individual’s constitution, also supports an approach in which treatment takes account of characteristics of the person rather than disease alone [50]. Classical discussions of the ideal Ayurvedic physician similarly combine knowledge and clinical skill with moral conduct and compassion [51]. These principles should not be taken to imply that Ayurvedic practice has always been uniformly compassionate or person-centred; rather, they identify ethical ideals expressed within particular Ayurvedic texts and traditions.

Compassion also has an important place in Buddhist ethics. Practices associated with *mettā* (loving-kindness) and *karuṇā* encourage concern for the well-being of others, including those experiencing illness [52]. These ideas have some parallels with contemporary approaches to compassionate and person-centred care, although the underlying philosophical traditions differ.

Modern clinical ethics has developed its own substantial traditions of beneficence, non-maleficence, autonomy, justice, empathy, and compassionate care [53]. Contemporary medicine also increasingly treats empathy as a quality that can be developed through education and clinical training [54]. The distinction, therefore, is not between an empathetic ‘East’ and a ‘West’ lacking in empathy. A more useful comparison concerns different ways traditions conceptualise and articulate empathy and compassion. In some Ayurvedic and Buddhist accounts, compassion forms part of the ethical character expected of the practitioner; in contemporary clinical practice, empathy may additionally be defined, taught, assessed, and strengthened as a professional competency [53,54]. Bringing these perspectives into dialogue offers global health a broader basis for considering how compassionate care can be supported in both individual practice and health professional education.

### Empathy as a link between cure and care

Empathy can help bridge the technical dimensions of clinical treatment with the relational dimensions of care. Biomedical knowledge and clinical intervention remain fundamental to effective treatment, while insights from diverse philosophical and ethical traditions can further reinforce how care, suffering and therapeutic relationships operate in unity. Empathy fosters trust and strengthens the therapeutic alliance, helping patients feel understood and respected, which allows for a candid dialogue and better compliance [55]. Meta-analyses and systematic reviews have shown that higher empathy in clinicians brings better outcomes: better symptom control, fewer complications, higher patient satisfaction and quality of life [56].

Practices informed by Eastern philosophy can also cultivate empathetic clinicians. For example, a meta-analysis on mindfulness-based interventions found statistically significant gains in empathy, indicating meaningful potential for application within clinical training programs [57]. Loving-kindness meditation (*mettā*) has similarly demonstrated a rise in positive emotions towards others and reduced implicit bias, offering indiscriminate care [58]. Remarkably, elements of Eastern approaches are increasingly growing within Western medical training too. Training in communication skills, an enhanced understanding of the doctor-patient relationship, and humanistic care curricula have emerged as formal attempts to incorporate the empathetic frameworks that have long been intrinsic to Buddhist contemplative traditions.

The analysis presents a clear case calling global health researchers to incorporate Eastern/Asian empathetic frameworks as a core element within mainstream global health discourse and extant literature.

### Implications for global health

Greater engagement with diverse philosophical and knowledge traditions has practical implications for how knowledge is produced, taught, applied and governed in global health (**Figure 1**).

**Figure 1:**
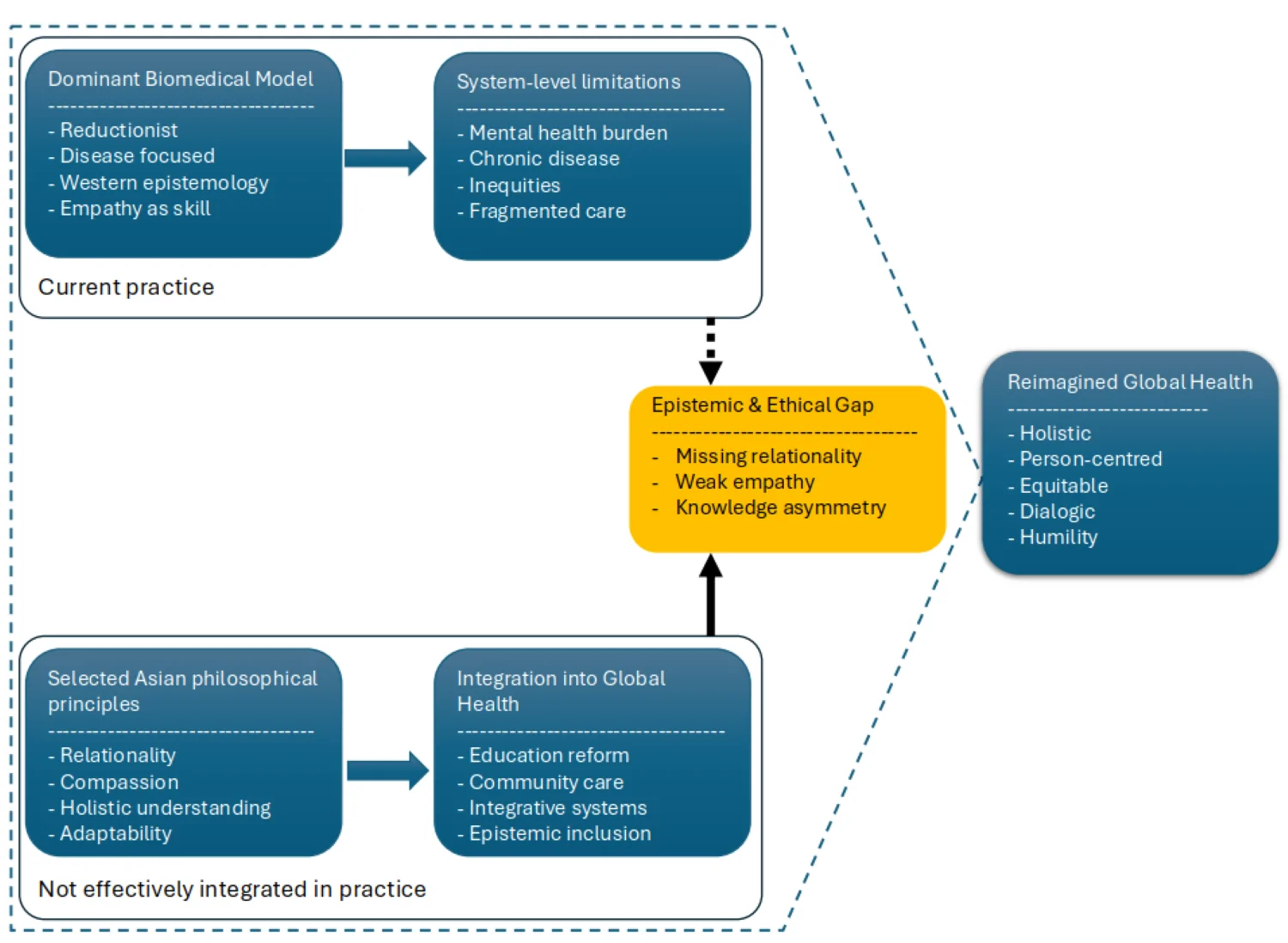
Implications of integrating selected Asian philosophies in contemporary global health.

First, it calls for a reorientation of medical and public health education, where empathy, relational awareness, and reflective practice are cultivated as foundational competencies rather than as complementary skill sets. In practice, curricula could expose students to different philosophical understandings of health, suffering and care alongside biomedical perspectives – for example, using Buddhist concepts of mindfulness and compassion or Ayurvedic understandings of person-centred care as opportunities for critical comparison and reflection. This does not imply embracing these traditions in their entirety or without critically challenging them, but rather carefully considering and qualifying their relevance to care, enabling future practitioners to recognise that patients and communities may understand health through diverse epistemological frameworks that extend beyond biomedical categories.

Second, knowledge pluralism has implications for how global health research questions are framed, and evidence is generated. Research could move beyond using local knowledge primarily to facilitate recruitment or intervention delivery and instead examine how communities themselves conceptualise health, suffering, recovery and well-being [59]. For example, qualitative and community-engaged research could identify locally grounded concepts that are not adequately captured by predetermined biomedical outcomes, and these could inform the questions asked, outcomes measured and interpretation of findings. Reflexivity would therefore extend beyond researchers examining their own positionality to questioning which forms of knowledge are recognised as legitimate evidence in the first place.

Third, health policies could become more epistemically responsive by recognising that communities may define health priorities, illness and acceptable care differently from biomedical institutions. Policy development could therefore incorporate deliberative processes in which community knowledge, lived experience and locally rooted understandings of well-being are considered alongside epidemiological and clinical evidence. For example, mental health programmes might assess not only symptom reduction but also outcomes that communities themselves associate with well-being, such as social connectedness, functioning, relational harmony or the ability to participate meaningfully in family and community life.

Finally, knowledge pluralism implicates who participates in global health decision-making and whose knowledge is regarded as authoritative. Advisory groups, guideline panels and research-priority-setting processes could include not only biomedical and technical experts but also scholars of relevant philosophical and knowledge traditions, practitioners, community representatives and people with lived experience. Their role should extend beyond consultation to contributing to how problems are defined, what outcomes matter and how evidence is interpreted. This would move pluralism from symbolic representation towards epistemic participation in decision-making, which could, at the very least, engender epistemic humility among scholars.

Knowledge pluralism, therefore, does not simply mean adding Eastern/Asian concepts to an existing global health framework. It requires openness to the possibility that engagement with different knowledge traditions may change the questions global health asks, the outcomes it values, the knowledge it considers legitimate, and who has authority to interpret that knowledge. Such dialogue need not diminish biomedical evidence; rather, it can broaden the epistemic resources through which complex and context-dependent problems of health and well-being are understood.

## CONCLUSIONS

We argue that greater engagement with diverse philosophical and knowledge traditions can broaden how health and well-being are understood and practised in global health. While biomedical advances remain indispensable, selected Asian philosophical and medical traditions offer complementary perspectives on relationality, compassion, adaptability and holistic care. The incorporation of mindfulness into contemporary healthcare provides one example of how concepts and practices originating in particular traditions can enter dialogue with biomedical and psychological approaches. Broadening global health knowledge therefore requires moving beyond one-directional knowledge transfer towards a more pluralistic and dialogic framework in which different knowledge traditions can contribute to the ethical, relational and systemic dimensions of health and well-being.

## Additional material


Online Supplementary Document

## Data Availability

**Data availability:** No data other than those presented within the manuscript and supplementary files were generated during the writing of this viewpoint.
