# Supplementary material for: Broadening global health knowledge: epistemic pluralism through Asian philosophical traditions
Source: J Glob Health. 2026 Nov 13;16:03038. doi: 10.7189/jogh.16.03038 (PMC13761194; doi:10.7189/jogh.16.03038)
Supplement: Supplementary file 1 [file jogh-16-03038-s001.pdf]

Supplement to: Hewage B, Joshi B, Poudyal Y, Banik S, Hanbookunuparkarn B, Ishioka H, Ding J, Acharya S, Mishra SR, Adhikari B. Broadening global health knowledge: epistemic pluralism through Asian philosophical traditions. J Glob Health. 2026;16:03038.

File S1. Chronological development of eastern philosophies

The evolution of the traditional texts referenced here reflects a long and layered intellectual history in which philosophical, medical, ethical, and contemplative traditions developed across South Asia, East Asia, and Tibet. Although these traditions emerged in distinct cultural contexts, they share recurring commitments to holism, relationality, and the cultivation of moral and cognitive discipline. Their development spans more than three millennia, beginning with early Vedic cosmology and extending through classical Buddhist, Confucian, Daoist, Ayurvedic, Chinese, Tibetan, and Unani textual traditions.

The earliest foundations lie in the Vedic corpus (c. 1500–500 BCE), which established ritual, cosmological, and proto-medical frameworks in ancient India (**Table S1**). While the Vedas themselves are not systematic medical manuals, they articulated a vision of cosmic order (*ṛta*) and interconnectedness that later informed both philosophical and medical traditions. Out of this milieu emerged the Upanishads (c. 800–300 BCE), which shifted emphasis from ritual performance toward introspective inquiry into consciousness, selfhood, and ultimate reality (Brahman–Ātman). These texts laid conceptual groundwork for meditative disciplines and for later formulations of Yoga and Advaita Vedānta, traditions referenced in the manuscript as cognitive and contemplative technologies aimed at mental equilibrium.

Parallel developments occurred in the so-called Axial Age (c. 700–300 BCE), during which several religious traditions emerged. In northern India, early Buddhism arose in the 5th century BCE. The Buddha's teachings were transmitted orally and later compiled into the Pāli Canon, with written redaction occurring around the 1st century BCE. Canonical discourses such as the Satipaṭṭhāna Sutta and the Anattalakkhaṇa

Sutta articulated foundational doctrines: *anicca* (impermanence), *anattā* (non-self), *dukkha* (suffering), and the cultivation of *sati* (mindfulness). These texts framed human suffering not merely as physical ailment but as existential misapprehension rooted in attachment and ignorance. Meditation practices such as *Vipassanā* (insight) and the cultivation of *mettā* (loving-kindness) and *karuṇā* (compassion) were systematized as disciplined methods for ethical and psychological transformation. In this tradition, liberation and well-being were inseparable from insight into the dynamic and contingent nature of experience.

At roughly the same historical moment in China, Confucian and Daoist traditions took shape. The Analects, compiled in the 5th–3rd centuries BCE, recorded the teachings of Confucius and emphasized moral cultivation, relational ethics, and social harmony. Health and flourishing were understood not in isolation but within properly ordered human relationships governed by ritual propriety and humane virtue. In contrast—but not opposition—Daoism (traditionally attributed to Laozi, 4th–3rd century BCE) articulated a cosmological and metaphysical vision centered on the Dao, the ineffable generative principle underlying reality. The principle of *wu wei* (effortless action) counseled alignment with natural processes rather than forceful intervention. The Zhuangzi further elaborated this orientation, advocating spontaneity, adaptability, and attunement to change. Together, Confucian and Daoist texts established complementary visions: one oriented toward ethical cultivation within social order, the other toward harmonization with cosmic process.

From these philosophical matrices developed structured medical traditions. Classical Ayurveda emerged between roughly 400 BCE and 200 CE, with the Charaka Saṃhitā and Sushruta Saṃhitā serving as foundational compendia. These texts codified diagnostic principles, dietary and lifestyle regulation, surgical procedures, and ethical guidelines for physicians. Central to Ayurveda was the concept of *prakṛti*—individual constitutional balance—along with the understanding that illness arises from disharmony among bodily, mental, and environmental factors. Notably, the ethical character of the healer was inseparable from clinical competence; compassion and

clarity of mind were codified as essential virtues. The holistic orientation of Ayurveda thus integrated physiology, psychology, and moral philosophy.

Between the 2nd and 8th centuries CE, Tibetan medicine also synthesized influences from Indian Ayurveda, Chinese medicine, and Buddhist psychology. Tibetan medicine integrated somatic diagnostics with spiritual and ethical considerations, reinforcing the premise that bodily equilibrium and mental clarity are interdependent. In this system, medicine functioned within a broader soteriological framework derived from Buddhist philosophy.

Meanwhile, in the Islamic Golden Age (8th–12th centuries CE), Greco-Arabic medical knowledge was preserved and systematized in works such as Ibn Sina's Canon of Medicine. This tradition, later known as Unani medicine in South Asia, emphasized humoral balance, dietary regulation, and ethical physician conduct. Although drawing from Hippocratic and Galenic sources, its development in Arabic and Persian intellectual contexts embedded it within broader Islamic philosophical traditions that valued harmony between body and spirit.

From the medieval period onward, Mahāyāna Buddhism evolved in East Asia into Chan (Zen in Japan), where meditation manuals and commentarial literature refined contemplative techniques emphasizing non-attachment and direct experiential awareness. Zen textual traditions continued the Buddhist emphasis on insight into impermanence and non-self, while adapting these teachings to new cultural contexts.

The late twentieth century witnessed a new phase in the transmission of these traditions through their clinical translation in Western contexts. Mindfulness-based interventions such as MBSR (1979) and MBCT (early 2000's) drew selectively from early Buddhist contemplative practices while reframing them within biomedical and psychological paradigms. Although these programs represent modern developments rather than ancient texts, they trace their conceptual lineage directly to the canonical discourses and meditative traditions outlined above.

Thus, the evolution of these Eastern traditional texts reflects neither a single linear progression nor a monolithic worldview. Instead, it reveals a constellation of traditions that emerged across multiple civilizations, converging around shared concerns: how to

live in harmony with change, how to cultivate ethical responsiveness, and how to understand health as an integrated state of balance. Their enduring influence suggests that these texts were not merely speculative philosophy but practical guides to embodied life—texts that continue to shape contemporary discourse precisely because they were always oriented toward the lived realities of suffering, healing, and human flourishing.

Table S1. Chronological development of eastern philosophies

**Period**	**Philosophical traditions**	**Key developments and concepts**
**Vedic period (c. 1500–500 BCE)**	Early Vedic traditions (India)	Development of cosmology, ritual traditions, and moral order linked to *dharma* and social harmony.
**Late Vedic / Upanishadic period (c. 800–300 BCE)**	Upanishads, Vedanta	Shift from ritual performance toward introspection, consciousness, selfhood, and ultimate reality (*Brahman–Atman*). Foundations of yoga and Advaita Vedanta emerge.
**Axial Age (c. 700–300 BCE)**	Buddhism, Jainism, Confucianism, Daoism	Emergence of major eastern philosophical traditions emphasizing ethics, compassion, suffering, mindfulness, social harmony, and moral cultivation.
**Classical Buddhist period (c. 500 BCE onward)**	Buddhism	Development of doctrines such as impermanence (*anicca*), non-self (*anatta*), suffering (*dukkha*), and cultivation of wisdom and compassion. Liberation and well-being viewed as

		inseparable from insight into human interdependence.
**Classical Chinese philosophy (c. 500–200 BCE)**	Confucianism and Daoism	Emphasis on relational ethics, collective harmony, virtue cultivation, balance with nature, and adaptive governance.
**Classical Ayurvedic period (c. 400 BCE onward)**	Ayurveda	Integration of body, mind, environment, and balance into holistic understandings of health and well-being.
**Medieval period (c. 500–1500 CE)**	Buddhist, Hindu, Daoist, and Islamic philosophical scholarship	Expansion of metaphysics, ethics, medicine, contemplative traditions, and knowledge systems across Asia.
**Modern and contemporary period**	Eastern philosophical revival and global influence	Increasing engagement with mindfulness, holistic health, relational ethics, systems thinking, decolonial scholarship, and knowledge pluralism in global health and social sciences.
